# Starvation ketoacidosis associated with tirzepatide use for weight loss in a non-diabetic East Asian woman: a case report

**DOI:** 10.1186/s12245-026-01170-9

**Published:** 2026-03-16

**Authors:** Yuki Minoda, Syuhei Ikeda, Koichi Inukai, Suguru Kishitani, Takamasa Takeuchi, Hirohiko Aikawa, Ken Saito, Hirotada Kittaka, Yasuyuki Hayashi, Hirotaka Sawano, Yusuke Ito

**Affiliations:** Senri Critical Care Medical Center, Saiseikai Senri Hospital, Osaka, Japan

**Keywords:** Tirzepatide, Mounjaro, GIP/GLP-1 receptor agonist, Starvation ketoacidosis, obesity, East Asian, Weight loss, Adverse drug reaction, Non-diabetic patient, Caloric restriction

## Abstract

**Background:**

Tirzepatide, a dual glucose-dependent insulinotropic polypeptide and glucagon-like peptide-1 receptor agonist, is increasingly used for obesity treatment, including in individuals without diabetes. Starvation ketoacidosis is a rare but serious complication associated with reduced caloric intake and enhanced ketogenesis. Reports of tirzepatide-associated starvation ketoacidosis in non-diabetic patients remain limited, and none have been reported in East Asian populations.

**Case presentation:**

A 21-year-old Japanese woman with obesity initiated weekly subcutaneous tirzepatide (2.5 mg/week) for weight loss while practicing carbohydrate restriction. After the fifth injection, she developed persistent nausea and vomiting, leading to markedly reduced oral intake. She presented to the emergency department with hypoglycemia (68 mg/dL) and severe high–anion gap metabolic acidosis (pH 7.22, bicarbonate 10 mmol/L, anion gap 22.6 mmol/L). Urinalysis revealed ketonuria, and serum ketone levels were markedly elevated. She had lost 21 kg one month after the initiation of tirzepatide. No infection, toxin exposure, or alternative metabolic disorder was identified. Starvation ketoacidosis was diagnosed. Treatment with intravenous glucose-containing fluids resulted in rapid resolution of metabolic acidosis within 12 h without insulin therapy.

**Conclusions:**

This case demonstrates that tirzepatide can precipitate starvation ketoacidosis in non-diabetic individuals, particularly when gastrointestinal adverse effects or dietary restriction lead to caloric deprivation. As the first reported case in an East Asian patient, it highlights the need for emergency physicians to consider starvation ketoacidosis in patients using tirzepatide for weight loss who present with vomiting, poor intake, or rapid weight loss. Early recognition and appropriate metabolic assessment are essential, and in patients with preserved endogenous insulin secretion, prompt glucose administration may allow rapid recovery without need for insulin therapy.

## Background

Tirzepatide is the first long-acting dual agonist of the glucose-dependent insulinotropic polypeptide (GIP) and glucagon-like peptide-1 (GLP-1) receptors. It received regulatory approval in the United States for the treatment of type 2 diabetes mellitus (T2DM) in 2022 and for obesity in 2023 [1], and in Japan tirzepatide was approved for T2DM in 2022 and for obesity disease in 2024, with commercial availability beginning in April 2025 [2]. Tirzepatide has been shown to induce greater weight loss than GLP-1 receptor agonists alone [3, 4]. The use of anti-obesity pharmacotherapies has increased rapidly, including among individuals without diabetes, partly driven by online prescription services [5].

Starvation ketoacidosis is a form of high-anion gap metabolic acidosis that occurs in the setting of severe caloric deprivation. When glycogen stores become depleted, lipolysis and hepatic ketogenesis are enhanced, resulting in excessive accumulation of ketone bodies. Ketoacidosis is biochemically characterized by elevated blood β-hydroxybutyrate levels (≥ 3.0 mmol/L), in the presence of metabolic acidosis [6, 7].

Recent reports have described ketoacidosis related to GLP-1 receptor agonists and GLP-1/GIP receptor agonists, particularly when used for weight loss in non-diabetic individuals [7, 8]. However, reported cases remain rare, and none have involved East Asian patients. We report a case of tirzepatide-associated starvation ketoacidosis in a non-diabetic young woman, highlighting diagnostic considerations in the emergency department.

## Case presentation

A 21-year-old Japanese woman with obesity (weight 75 kg, body mass index [BMI] 30.2 kg/m²) initiated carbohydrate restriction for weight loss and began weekly subcutaneous injections of tirzepatide 2.5 mg prescribed through an online clinic one month prior to presentation. Approximately four days before admission, after her fifth injection, she developed persistent nausea and vomiting that progressively limited her oral intake. Her symptoms did not improve, and she developed worsening generalized fatigue, prompting presentation to the emergency department.

She had no history of diabetes mellitus. Her medications included weekly tirzepatide 2.5 mg, metoclopramide 5 mg once daily, and vonoprazan fumarate 10 mg once daily. She was not taking herbal medicines or dietary supplements and reported no alcohol consumption. In Japan, tirzepatide use for obesity under insurance requires registered dietitian counseling; however, because the patient obtained it online, she did not receive formal nutritional guidance.

On arrival, she was alert. Her vital signs were as follows: blood pressure 107/77 mmHg, heart rate 108 beats/min (regular), respiratory rate 12 breaths/min, temperature 36.1 °C, and oxygen saturation 98% on room air. Her height was 157 cm and weight 54 kg (BMI 21.9 kg/m²), indicating a weight loss of 21 kg within one month. Physical examination revealed no conjunctival pallor or jaundice, no dry mucous membranes, no decreased skin turgor, a soft and non-tender abdomen, normal heart and breath sounds, and no peripheral edema. Given the history of repeated vomiting and the need to exclude structural gastrointestinal pathology, non-contrast-enhanced abdominal computed tomography was performed. No gastrointestinal abnormalities were identified; however, collapse of the inferior vena cava was noted, suggesting volume depletion.

Laboratory evaluation showed hemoconcentration on complete blood count. Venous blood gas analysis revealed pH 7.22, bicarbonate (HCO_3_^−^) 10 mmol/L, base excess − 15.5 mmol/L, anion gap 22.6 mmol/L, and glucose 68 mg/dL, consistent with high–anion gap metabolic acidosis with hypoglycemia (Table 1).

**Table 1:** Laboratory results

Biochemistry		Complete blood count		Venous blood gas analysis (room air)	
AST	25 U/L	WBC	8.8 × 10^3^ /µL	pH	7.22
ALT	14 U/L	RBC	5.89 × 10^6^ /µL	HCO_3_^−^	10 mmol/L
BUN	16 mg/dL	Hb	17.5 g/dL	Base excess	-15.5 mmol/L
Cre	0.7 mg/dL	Ht	51.9 %	Anion Gap	22.6 mmol/L
Na	136 mmol/L	PLT	307 × 10^3^ /µL	Lactate	1.4 mmol/L
K	4.5 mmol/L			Glucose	68 mg/dL
Cl	105 mmol/L	**Urinalysis**			
IP	3.2 mmol/L	Glucose	(-)		
Mg	2.2 mg/dL	pH	5		
		Ketone	(3+)		

Abbreviations: HCO_3_^−^, bicarbonate; AST, asperate aminotransferase; ALT, alanine aminotransferase; BUN, blood urea nitrogen; WBC, wihite blood cell count; RBC, red blood cell count; Hb, hemoglobin; Ht, hematocrit

Urinalysis demonstrated ketonuria. Given the combination of severe high–anion gap metabolic acidosis and hypoglycemia in a patient without diabetes, starvation ketoacidosis was promptly suspected in the emergency department. Additional biochemical testing revealed markedly elevated total ketone bodies, acetoacetic acid, and β-hydroxybutyrate (Table 2).

**Table 2:** Fasting biochemical findings (Additional Laboratory Results)

Insulin	16.4	µU/mL	(Reference range: 1.1-17.0 µU/mL)
C peptide	3.1	ng/dL	(Reference range: 1.1-3.3 ng/mL)
HbA1c (NGSP)	4.9%		(Reference range: 4.6-6.2%)
Total ketone bodies	7,410	µmol/L	(Reference range: 0-131 µmol/L)
Acetoacetic acid	1,516	µmol/L	(Reference range: 0-55 µmol/L)
β-Hydroxybutyrate	5,894	µmol/L	(Reference range: 0-85 µmol/L)

**Fig. 1:**
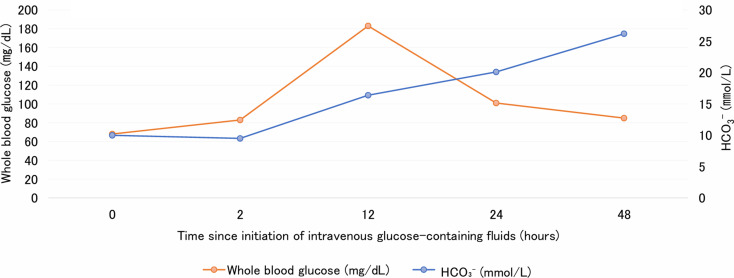
Time course of blood glucose and bicarbonate (HCO_3_^−^) levels after initiation of intravenous glucose-containing fluids

The patient was treated with intravenous acetated Ringer’s solution containing 5% glucose. Metabolic acidosis improved rapidly and resolved within 12 h of treatment (Table 3; Fig. 1). Insulin therapy and electrolyte supplementation were not required. By hospital day 3, her gastrointestinal symptoms had resolved, and she was able to tolerate oral intake. She was discharged in stable condition.

**Table 3:** Timeline of laboratory findings

Venous blood gas analysis	Clinical Course After Presentation (Hours)			
(room air)	0	2	12	24
pH	7.22	7.25	7.42	7.38
HCO_3_^−^ (mmol/L)	10	9.5	16.4	20.1
Anion Gap (mmol/L)	22.6	19.7	7.7	9.2
Lactate (mmol/L)	1.4	1.1	1.3	1.3

Abbreviation: HCO_3_^−^, bicarbonate

## Discussion

GIP and GLP-1 are incretin hormones that stimulate glucose-dependent insulin secretion. Tirzepatide acts on both receptors and enhances insulin secretion in a glucose-dependent manner. In addition, it suppresses appetite, reduces caloric intake, and delays gastric emptying, and is frequently associated with gastrointestinal adverse effects such as nausea and vomiting [8–10]. Severe carbohydrate restriction and reduced caloric intake may lead to decreased insulin secretion and relative glucagon predominance, promoting lipolysis and hepatic ketogenesis [7]. In susceptible individuals, this metabolic shift can result in starvation ketoacidosis even in the absence of diabetes.

In the present case, a combination of intentional carbohydrate restriction and severe gastrointestinal adverse effects likely resulted in profound caloric deprivation, precipitating starvation ketoacidosis. Importantly, metabolic acidosis resolved with glucose-containing fluids alone, without continuous intravenous insulin infusion. This clinical course suggests that endogenous insulin secretion was preserved. Given tirzepatide’s glucose-dependent insulinotropic effect, insulin secretion may not have been markedly suppressed despite reduced caloric intake. Therefore, a detailed medication history, including recent use of GLP-1 receptor agonists and GIP/GLP-1 receptor agonists, is essential in the emergency settings. In non-diabetic patients with preserved insulin secretion, treatment with glucose-containing fluids alone may be sufficient to suppress ketogenesis.

A literature review identified nine previously reported cases of ketoacidosis associated with tirzepatide or other GLP-1 receptor agonists in non-diabetic patients [11–16] (Table 4).

**Table 4:** Reported cases of EKA in non-diabetic patients associated With Tirzepatide and GLP-1 receptor agonists(GLP-1 RAs)

Year	Author	Age (y.o)	Sex	BMI (kg/m2)	Medication	Duration of Treatment	Blood Glucose (mg/dL)	Treatment	Outcome
2022	Alghamdi et al. [11]	27	Female	30	GLP-1RAs	10 days	normal	glucose-containing fluids, insulin	alive
2023	Mercer et al. [12]	29	Female	26.5	tirzepatide	3 weeks	86	glucose-containing fluids	alive
2024	Iqbal et al. [13]	29	Female	28.2	tirzepatide	3 weeks	88.2	glucose-containing fluids	alive
2024	Sood et al. [14]	29	Female	31	GLP-1RAs	7 months	107	glucose-containing fluids	alive
2024	Bitar et al. [15]	29	Female	32	tirzepatide	7 weeks	54	glucose-containing fluids	alive
2024	Bitar et al. [15]	29	Female	31.3	tirzepatide	6 weeks	50.4	glucose-containing fluids	alive
2024	Bitar et al. [15]	29	Female	30.4	tirzepatide	5 weeks	52.2	glucose-containing fluids	alive
2024	Bitar et al. [15]	29	Female	30.8	tirzepatide	6 weeks	55.8	glucose-containing fluids	alive
2025	Singh et al. [16]	29	Female	30.4	tirzepatide	one month	104.4	fluids	alive
2025	Our case	21	Female	30.2	tirzepatide	one month	68	glucose-containing fluids	alive

Abbreviation: GLP-1, glucagon-like peptide-1

All cases occurred in women using these agents for weight loss, and gastrointestinal symptoms were common preceding features. Most cases improved with intravenous glucose-containing fluids alone, supporting conservative management in appropriately selected patients.

To our knowledge, this is the first reported case of tirzepatide-associated starvation ketoacidosis in an East Asian individual. East Asians generally have a higher body fat percentage and lower skeletal muscle mass than individuals of similar BMI from other ethnic groups, potentially resulting in lower total glycogen reserves [17]. This finding raises the possibility that such metabolic characteristics may predispose East Asian patients to more rapid glycogen depletion and earlier ketosis during periods of caloric deprivation. This hypothesis should be considered exploratory and warrants further investigation.

Given tirzepatide’s long half-life of approximately 5–6 days, sustained receptor activation may contribute to prolonged metabolic effects even after drug discontinuation [9, 18]. Emergency physicians should remain vigilant for starvation ketoacidosis in patients using tirzepatide for weight loss, particularly those presenting with vomiting, poor intake, or rapid weight loss.

## Conclusions

Tirzepatide can precipitate starvation ketoacidosis in non-diabetic individuals when caloric intake is markedly reduced due to gastrointestinal adverse effects or dietary restriction. In patients with preserved endogenous insulin secretion, glucose-containing fluids alone may be sufficient for rapid metabolic recovery without insulin infusion. Emergency physicians should consider starvation ketoacidosis in patients receiving tirzepatide who present with high–anion gap metabolic acidosis despite hypoglycemia. Early recognition and prompt glucose administration can lead to rapid recovery.

## Data Availability

No datasets were generated or analysed during the current study.
